# Supplementary material for: Are carbon emissions trading and green financial instruments synergistic? -Comprehensive quantitative research based on content analysis
Source: PLoS One. 2024 Mar 7;19(3):e0298601. doi: 10.1371/journal.pone.0298601 (PMC10919601; doi:10.1371/journal.pone.0298601)
Supplement: S1 Data — (ZIP) [file pone.0298601.s001.zip › Data/地级市Z_F工作报告.zip!/╔╜╬≈/│ñ╓╬╩╨/└┤╫╘╣1⁄2╓┌║┼í╛┬φ┐╦╩2╛▌═°í┐_2011.docx]

各位代表：

　　现在，我代表市人民政府向大会作工作报告，并就《长治市国民经济和社会发展第十二个五年规划纲要（草案）》作说明，请予审议，请市政协委员和其他列席人员提出意见。

　　一、“十一五”时期国民经济和社会发展回顾

　　“十一五”时期是我市发展史上极不平凡的五年。五年来，面对经济结构的深层次矛盾特别是国际金融危机的严重冲击，我们在市委的领导下，在市人大、市政协的监督支持下，深入贯彻落实科学发展观，紧紧围绕党的十七大和十七届三中、四中、五中全会精神以及省委、省政府转型跨越发展的工作部署，团结带领全市广大干部群众，聚精会神搞建设，一心一意谋发展，千方百计促和谐，经济社会发展和各项事业都取得了新的成就，圆满完成了“十一五”规划目标任务。

　　（一）综合经济实力显著增强。5年全社会固定资产投资完成1686.5亿元，年均增长21%。特别是2009年以来应对国际金融危机、实施“双十工程”完成投资1004.7亿元，两年投资是“十五”时期的1.7倍，占“十一五”投资总额的60%；社会消费品零售总额年均增长20.6%；进出口总额年均增长17%。2010年，全市地区生产总值完成920.2亿元，是2005年的2.3倍；财政总收入198亿元，是2005年的2.8倍；一般预算收入77.9亿元，是2005年的3.3倍；城镇居民人均可支配收入17123元，农民人均纯收入5960元，分别比“十五”末增长87.6%和66.8%。经济总量由“十五”末的全省第四位跃居第二位。

　　（二）转型发展迈出坚实步伐。加快推进工业新型化，煤基合成油、太阳能光伏、光电子、微车制造、燃油乳化等战略性新兴产业快速崛起，全国第一桶具有完全自主知识产权的煤基合成油成功出油，全球第一条100万伏特高压输变电线路并网运行，全国惟一垂直整合的LED全产业链加快建设并初具雏形。下大力气改造提升煤、焦、冶、电等传统产业，义无反顾推进煤炭资源整合煤矿兼并重组，煤炭机械化开采率达到100%，整合重组工作在全省名列前茅。大力发展循环经济，形成煤化工、硅工业、LED、镁工业、乙炔化工、玉米深加工6条循环产业链，被列为全省循环经济试点市，被科技部等十九部委确定为全省惟一的国家可持续发展实验市。加快发展现代农业，旱作节水、设施蔬菜、机械化保护性耕作成效显著，农业综合生产能力稳步提高，粮食总产量稳定在14亿公斤左右，农业产业化龙头企业达到289个，农产品加工转化率达到45%，成为全国第一个农业综合标准化示范市。大力推进现代服务业，浦发银行、邮政储蓄银行、大同证券落户我市，组建了长子县村镇银行及一批小额贷款公司，开工建设了长治物流中心，被商务部确定为流通领域现代物流示范市，成功举办了国际攀岩节，荣获中国优秀旅游城市、中国旅游品牌十大目的地称号。

　　（三）城镇特色化快速推进。编制实施了《上党城镇群规划》及行动纲要，建成了115公里、全省最长的环城高速路，完成了天然气入市管网工程，“1+6”上党城镇群建设步伐不断加快。新区起步区路、水、电等基础设施工程进展顺利。建成了客运东站、体育中心、六府塔等一批标志性工程。启动实施了大县城、重点镇、中心村建设，进一步强化了城乡基础设施和公共服务能力。认真开展城乡环境卫生集中整治，全市城市生活垃圾无害化处理率和街道清扫率均达到100%，成为全省惟一的国家卫生城市，全省10个国家卫生县城有8个在我市，是全省国家卫生县城最多的市。

　　（四）城乡生态环境持续改善。实施了百家企业节能行动和十大节能重点工程，5年累计淘汰落后产能1578万吨。初步核算，万元地区生产总值能耗累计下降26.9%，成为“十一五”全省能耗降幅最大的市。深入实施“蓝天碧水工程”和“两地”保护工程，启动了浊漳河流域综合治理工程，二氧化硫和化学需氧量排放量分别比2005年下降14.3%和12.1%，超额完成“十一五”减排任务。全面推进造林绿化，5年完成造林200万亩，森林覆盖率由26.9%提高到30.9%，在我市召开了全国造林绿化现场会，荣获国家园林城市、全国绿化模范城市和全省造林绿化突出贡献奖。市区空气质量二级以上天数达到356天，比2005年增加91天，在国家环保部重点监测的城市中排名由81位上升到17位，连续7年成为全省空气质量最好的城市，连续3年名列华北第一，5个县荣获省级环保模范城市称号，形成了全省首个环保模范城市群。

　　（五）安全发展水平稳步提升。被国家安监总局确定为全国惟一的本质安全型城市试点市，建立了矿井机械化改造、安全隐患当事故处理、煤矿企业全员安全培训等制度。搞好煤矿整合重组期间的安全生产，实现了奥运期间煤矿不停产、零事故。深入推进安全生产和社会稳定“双排查”，狠抓安全生产专项整治。全市248个高危企业全部建立了安全生产指挥中心，形成了以安全为中心的企业运行新机制。2010年全市亿元生产总值死亡率比2005年下降75.4%，地方煤矿百万吨死亡率比2005年下降97.3%，工矿企业连续八年没有发生死亡6人以上的重大事故，安全生产形势在全省率先实现稳定好转。

　　（六）改革开放取得丰硕成果。市县政府机构改革、文化广电体制改革全面完成，事业单位分类改革、集体林权制度改革、医药卫生体制改革进展顺利，财税体制改革、行政审批制度改革进一步深化。国有企业改革步伐加快，长钢与首钢成功重组，实现了我市钢铁产业发展的历史性突破。全市民营经济增加值占地区生产总值比重由“十五”末的52%提高到56.5%。对外开放水平不断提高，积极参加港洽会、中博会、能博会等大型招商活动，引进了荷兰飞利浦等世界500强企业和德国森特塞姆等知名企业，吸纳外来资金640多亿元，振东制药在创业板成功上市。与德国北威州玛尔市等国际友好城市的经济合作进一步加强，与沿海发达地区、环渤海经济圈、中原经济协作区的合作交流更加广泛。

　　（七）各项社会事业全面进步。大力实施“五大惠民工程”，在教育方面，义务教育阶段免学费政策扩大到公办职业高中和普通高中家庭困难学生，郊区、沁源和长治县实现了普通高中全免费；在就业方面，5年累计新增城镇就业18.5万人，城镇登记失业率控制在3%以内；在医疗方面，县乡村三级卫生机构达标率93.7%，新型农村合作医疗参合率93.8%；在社会保障方面，城镇养老、医疗、失业等社会保险覆盖率达到90%以上，城乡低保对象实现应保尽保，具备了农村五保户100%集中供养条件，实施了城镇居民医保制度，在全省率先实现“全民医保”；在住房方面，建设经济适用房、廉租房和棚户区改造住房254万平方米、3.2万套。省委、省政府安排的农村“五个全覆盖”全面完成，同步实施了我市农村“五个全覆盖”，完成1077个试点村和重点推进村新农村建设任务，连续三年受到省委、省政府表彰。人口计生、新闻出版、体育健身等工作都取得优异成绩，双拥共建、国防教育、民兵预备役、人民防空工作进一步巩固。老龄、妇女儿童、残疾人事业健康发展。文物保护、外事侨务、防震减灾、红十字会、慈善救助、气象、统计、档案、保密、地方志等各项事业全面发展。

　　（八）民主法制和精神文明建设扎实推进。市政府主动向市人大报告工作，认真执行市人大及其常委会的各项决议，积极支持人民政协工作，高度重视人大代表建议和政协委员提案办理工作，办复率达到100%。加强与各民主党派、工商联、无党派人士和人民团体的联系，广泛听取各方面的意见和建议。坚持依法行政，完成了“五五”普法，不断扩大政务公开范围，强化行政监察和审计监督，党风廉政建设和反腐败工作取得新成效。扎实推进“平安长治”建设，切实加强社会治安综合治理，严厉打击各类刑事犯罪活动，治安形势持续稳定。加强信访工作，及时解决广大群众的诉求。全面贯彻党的民族宗教政策，市政府被国务院表彰为全省惟一的全国民族团结进步模范集体。精神文明建设取得新进展，太行精神、纪兰精神更加深入人心，文明城市创建活动扎实开展，再次荣获创建全国文明城市先进城市称号。全力以赴支援四川汶川、青海玉树、甘肃舟曲抗震救灾，高标准完成了对口援建任务。加强应急管理，有效应对强降雪、大风、大旱等自然灾害，妥善处置问题奶粉、甲型H1N1流感重大突发事件，圆满完成北京奥运、国庆60周年安保任务，社会和谐稳定的局面更加稳固。

在这里，我向大会郑重报告，我市“十一五”目标任务圆满完成，向全市人民承诺的实事全部兑现！

　　各位代表，回顾这五年，我们深切地感到，办好长治的事情，必须以科学发展观为统领，统筹推进经济、政治、文化、社会和生态建设全面协调可持续发展；必须牢牢把握发展这个第一要务，凝聚各方智慧和力量，形成同心同德、共谋发展的强大合力；必须以改革创新精神完善发展思路，破解发展难题，激发转型跨越发展的动力和活力；必须把安全稳定作为一切工作的底线，推动科学发展，促进社会和谐；必须把改善民生作为为政之要，让人民群众共享改革发展成果，不断提升全市人民的幸福指数。这些经验是全市人民生动实践的集中概括，也是我们在今后工作中必须坚持的重要原则。

　　各位代表，成绩来之不易，经验弥足珍贵。我市“十一五”所取得的成就，是省委、省政府和市委正确领导的结果，是市人大、市政协有效监督、积极支持的结果，是全市广大干部群众团结奋斗、共同努力的结果。在此，我代表市人民政府，向广大工人、农民、知识分子和各级干部，向各民主党派、工商联、无党派人士和人民团体，向驻市部队广大指战员、武警官兵、公安干警，向所有关心支持长治发展的各界朋友，致以崇高的敬意和衷心的感谢！

　　在充分肯定成绩的同时，我们也清醒地看到，我市欠发达的基本市情没有改变，经济发展方式粗放，产业结构不合理，经济总量不大，项目储备不足，科技创新能力不强；城乡发展不协调，城市建设滞后，城镇化率较低；社会和民生欠账较多，优质教育、医疗资源总量不足；各级干部特别是领导干部思想认识、能力水平与转型跨越发展的要求还不相适应等。对于这些问题，我们将高度重视，认真加以解决。

　　二、“十二五”时期的指导思想和目标任务

　　“十二五”时期是我市转型跨越发展的攻坚时期，是推进国家资源型经济转型综合配套改革试验区建设的黄金时期，是全面建设小康社会的关键时期。根据市委九届十二次全会精神及“十二五”规划《建议》，市政府编制了“十二五”规划《纲要（草案）》，明确提出“十二五”时期要全面贯彻党的十七届五中全会和省委九届十一次全会精神，以邓小平理论和“三个代表”重要思想为指导，深入贯彻落实科学发展观，以科学发展为主题，以加快转变经济发展方式为主线，以转型跨越发展为主旋律，以国家资源型经济转型综合配套改革试验区建设为统揽，以工业新型化、农业现代化、市域城镇化和城乡生态化为载体，着力调整经济结构，着力保障和改善民生，着力建设资源节约型和环境友好型社会，着力改革开放，着力科技创新，把我市打造成全省乃至全国新兴能源产业基地和全省转型综改试验区的先行区，在转型跨越发展的伟大实践中重振上党雄风，再造一个新长治。

　　“十二五”时期经济社会发展的主要目标是：转型发展实现新突破、跨越发展跃上新台阶、新兴能源产业基地建设创造新典范、城乡建设达到新水平、改革开放迈出新步伐、人民生活得到新提高。到2015年，实现地区生产总值、全社会固定资产投资、财政总收入和一般预算收入、城乡居民收入翻一番。

　　具体完成好以下几项任务：

　　（一）大力实施项目带动战略，夯实转型跨越发展的基础。在城乡基础设施、产业升级改造、民生、住房保障等领域布局一批大项目、好项目。积极引进战略投资者，拓宽招商引资、信贷债券、上市融资等多种融资渠道，进一步优化投资结构，提高投资效益。健全统筹推进重大项目建设工作机制，实现项目建设新突破，积蓄发展新动力。

　　（二）强力推进“四化”建设，搭建转型跨越发展的新平台。加快工业新型化，坚持以煤为基、多元发展，改造提升传统产业，培育壮大新兴产业，加快发展现代服务业，形成产业集群优势，构建具有长治特色的现代产业体系。以规模化、机械化、安全化、集约化为方向，做足煤炭产业扩能增值和升级改造大文章；以上数量、上环保、上节能为重点，做足清洁能源和新能源发展大文章；以抓扩张、提档次、增份额为目标，做足推进转型工业项目大文章。到2015年煤炭产能由1亿吨提高到2亿吨，实际产量达到1.5亿吨,电力总装机容量提高到2400万千瓦，年发电量达到1400亿度，新兴产业增加值占工业增加值的比重由17%上升到50%以上，三次产业比例由2010年的4.4：65.4：30.2调整为5：55：40。加快农业现代化，以建设全国一流的高效生态农业示范区为目标，以增加农民收入为核心，培育壮大骨干龙头企业，到2015年形成30个销售收入超亿元的农产品加工龙头企业，全市农产品加工转化率达到65%以上；以“一县一业、一村一品”为抓手，建设优质玉米、小杂粮、设施蔬菜、食用菌、干鲜果、畜禽养殖6大高效特色产业板块，到2015年全市50%以上的行政村要成为“一村一品”专业村；不断强化科技服务装备支撑，到2015年建成100个现代农业科技示范园区，带动高效设施农业面积占到总播面积的10%，标准化生产覆盖率达到90%，农业科技进步贡献率达到55%，各类专业合作社发展到8000个，农户参加合作经济组织的比例达到80%，力争主要农作物生产综合机械化水平达到70%。加快市域城镇化，按照“一核双圈”城镇空间布局，形成主城区为核心，上党城镇群6个县市为内圈，其他5个县为外圈的城镇化体系。到2015年全市城镇化率达到55%，主城区人口达到80万人左右，建成中国最宜居城市；全面加快“1+6”上党城镇群内圈建设，城镇人口达到170万，打造全省一流的城镇群；外圈5个县要按照“一城三镇百村”布局，在抓好大县城建设的同时，每个县发展3个重点镇和100个中心村，县城人口规模达到10万人以上，重点镇人口达到1万人以上，中心村人口达到1000人以上，实现主城区、大县城、重点镇、中心村一体化发展。加快城乡生态化，按照重点主攻荒山、巩固提高平川的绿化思路，到2015年全市森林面积达到771万亩，森林覆盖率达到37%，建城区绿化覆盖率达到50%，努力构建现代林业生态体系。

　　（三）全力保障和改善民生，营造转型跨越发展的和谐环境。继续实施科教兴市、文化强市和人才强市战略，大力发展科技、教育、卫生等各项社会事业，提升文化软实力。千方百计扩大就业，完善社会保障体系，推进收入分配制度改革，加大保障性住房建设力度，努力使发展成果惠及全市人民。到2015年，全市城镇基本社会保障覆盖率达到80%，人均预期寿命达到75岁。全面加强社会管理，完善社会治安防控体系，改进信访工作，深入创建本质安全型城市，搞好安全生产工作。发展社会主义民主政治，建设社会主义精神文明，不断推进法治政府建设，促进经济社会协调发展。

　　（四）着力先行先试，走出转型跨越发展的新路子。要抓住并用好转型综改试验区这一重大机遇，用足用活试验区这个大品牌、大载体、大平台，强化组织领导，强化政策引导，强化保障支持，大力推进综合配套改革，促进经济社会转型跨越发展。营造体制机制新优势，以积极的姿态破解资金、土地、人才、技术、劳动力等瓶颈制约，为再造一个新长治扫清障碍、铺平道路。以更大的信心和勇气全面推进各项改革，力争在经济管理、行政管理和社会管理等重点领域和关键环节上取得新突破。大力开展招商引资，坚持领导带头抓招商、万众一心齐招商、瞄准高端招大商、创优环境引外商。各县市区主要领导每人每年至少引进一个投资在亿元以上的项目，每个县每年至少签订15个招商引资合同，项目落地率要达到70%以上。坚持招商引资和招才引智相结合，强化人才资源是第一资源的观念，培养、使用与引进并重，全面加强和创新人才工作。加强对外交流合作，强化与国内外大企业、大集团的合作，全面提升我市对外开放水平，以大开放促进大跨越。

　　各位代表，这些目标和任务综合考虑了省委、省政府的要求和全市人民的期盼，考虑了我市的发展基础条件和潜力优势，充分体现了转型跨越、创优争先、惠民利民的要求。我们一定要坚定信心、同心同德，勤勉前行、永不懈怠，让“十二五”规划的宏伟蓝图在上党大地变为壮丽的现实！

三、2011年的主要工作

　　2011年是全面实施“十二五”规划的开局之年，是推进国家资源型经济转型综合配套改革试验区建设的起步之年，做好今年工作意义重大，影响深远。今年全市经济社会发展的主要目标是：地区生产总值增长15%；全社会固定资产投资增长25%；社会消费品零售总额增长16%；财政总收入和一般预算收入均增长15%；城镇居民人均可支配收入、农民人均纯收入分别增长12%和12%以上;居民消费价格总水平涨幅控制在4%左右；城镇登记失业率控制在3%以内；万元地区生产总值综合能耗下降3.5%左右;万元地区生产总值二氧化碳排放量下降3.5%;二氧化硫、化学需氧量排放量分别下降1.5%和2%;氮氧化物、氨氮排放量均下降1.5%；烟尘、粉尘排放量均下降2%；万元工业增加值用水量下降5%。

　　围绕上述目标，今年要重点抓好以下九个方面工作：

　　（一）把项目建设作为经济工作第一抓手，促进经济快速健康发展

　　着力扩大项目投资。今年初步安排1057个项目，总投资2468亿元，年内计划完成投资805亿元。进一步优化投资结构，加大新兴产业、城市建设、民生等领域的投资，安排转型升级的产业项目409个，完成投资458亿元，占投资总额的56.9%；安排城镇化建设项目373个，完成投资271亿元，占投资总额的33.7%。重点要抓好20个非煤非电项目。通过加大项目投资，推动转型跨越发展。

　　着力强化项目管理。提高项目管理服务水平，形成部门协调联动、管理服务高效、组织实施规范的项目管理模式。成立推进重大项目建设领导组，建立市县领导帮办督办、观摩检查、督查通报和责任追究制度，实行“一个项目、一位领导、一套班子、一抓到底”的工作机制。严格执行项目法人制、招投标制、合同管理制、工程监理制和概算审计、跟踪审计、决算审计“四制三审”制度，抓开工、促在建、保质量、快投产。

　　着力加强项目储备。积极谋划、储备一批符合国家产业政策、管全局、利长远、带动性强的重大项目。强化项目库建设，将项目库储备的一万多个项目信息按行业和规模进行分类筛选、策划、包装，纳入项目管控一体化电子平台进行管理。抓好项目前期研究和规划设计，加快环境评价、土地预审等各种手续跑办进度，做好被征地群众的权益保护工作，保障项目顺利实施。

　　各位代表，我们要牢固树立抓项目就是抓发展的理念，充分发挥项目投资的拉动作用，以投资总量的增加和投资增量的扩张，全力保持经济发展的强劲势头，确保今年我市地区生产总值进入地级市“千亿元俱乐部”!

　　（二）以煤为基、多元发展，着力推进工业新型化

　　加快煤炭企业转型步伐。以整顿和规范煤炭行业秩序为重点，扎实做好煤炭工业改革发展各项工作。加快矿井升级改造步伐，进一步提高煤炭生产规模化、机械化、安全化、集约化水平。把煤炭产业做极致、做精细、做洁净，延长煤焦化、煤气化、煤液化、煤电化等产业链，积极发展下游产品。制定煤炭企业转型办法，纳入年度目标责任考核范围，所有煤矿主体企业都要在发展煤炭采掘洗选设备、煤矿安全设备、煤矿环保设备、煤基化工、煤炭物流以及新能源、新材料、节能环保、旅游服务业、文化产业和社会公益性事业等非煤产业方面有新举措，实现多元化发展，年内煤矿企业非煤产业的比重由现在的6%提高到15%。

　　加快构建现代产业体系。发挥潞安集团、首钢长钢、山西煤销长治公司、淮海、惠丰、清华等国省属企业的引领带动作用，促进市内企业转型发展。改造提升传统产业，推进焦化、冶金、电力等行业整合重组，焦炭产业以集群化为方向，提高产业集中度，重点建设潞安集团和潞宝集团两个7.63米超大型焦炉；冶金产业以大型化为方向，推动首钢长钢与市内6家钢铁企业的重组，把首钢长钢公司建成全国最大的棒材生产基地；电力行业以清洁化为方向，加快建设100万伏特高压二期工程，推进漳电2×100万千瓦发电机组、高河电厂、赵庄二期等电源点建设。培育壮大新兴产业，大力发展太阳能光伏、新材料、煤化工、装备制造、光电子、中西制药、食品加工、现代服务业等战略性新兴产业，重点推进LED全产业链光电子产业基地、成功淮海30万辆微车制造、潞安集团60万吨烯烃、潞城纳米碳增效肥、惠丰公司人工心脏等项目建设。加快建设达利休闲食品扩建、沁州黄农产品产业化开发、金泽玉米深加工、平顺20万亩中药材种植等项目，力争全市农副产品加工转化率达到50%以上。大力发展现代服务业和旅游文化产业，推进以长治现代物流中心为龙头的35个物流企业和物流园区建设。加快实施太行山大峡谷、平顺太行水乡、沁县莱茵湖畔旅游综合开发项目。实施文化强市战略，建设文化传媒创意园区和武乡八路军文化园区，打造城市文化产业集群，推动文化旅游一体化发展，把物流、旅游、文化产业培育成我市新的支柱产业。

　　加快新兴能源产业基地建设。编制《长治市新兴能源产业基地建设发展规划》，组建低碳研发中心、纳米研究院、光电研发中心和新能源工程技术中心，加快建设潞安集团1万吨多晶硅、黎城天源4万吨多晶硅、540万吨煤基合成油、100万吨燃油乳化、1GW太阳能电池板、壶关地下原位煤制气、沁新煤层气发电、平顺大唐风能太阳能发电、成功淮海60万台甲醇发动机等21个新兴能源项目，力争在新兴能源产业发展上有大作为、新突破。

　　加快发展循环经济。按照“多联产、全循环、抓高端”的思路和“减量化、再利用、资源化”的原则，全面推进各类产业特别是传统产业的循环化发展。提高煤矸石、粉煤灰、工业废渣、废水、废气的综合利用水平。搞好省级循环经济试点市的各项工作，重点抓好光电园区、武乡蟠洪工业园区和首钢长钢、襄垣煤矿等循环经济园区（企业）的循环发展，构建企业、行业、园区、社会、区域五个层面的循环经济体系，逐步形成企业小循环、园区中循环、社会大循环的良性互动格局。

　　各位代表，工业新型化是长治转型跨越发展的第一着力点，是重振上党雄风的第一推动力。我们要让煤焦产业浴火重生，让电力产业脱胎换骨，让新兴产业加速崛起，努力构建具有长治特色的现代产业体系！

　　（三）不断强化“三农”工作，加快推进农业现代化

　　大力发展高效特色农业。围绕六大高效特色产业板块，研究制定“一县一业、一村一品”发展和推进规划，提高特色优势农产品集聚度。搞好农业科技示范园区建设，进一步壮大方兴农业、紫团食用菌等设施农业生产基地，规范提升工厂化育苗的水平和质量。深入推进农业产业化，新上95个农业产业化项目，促进农业生产的专业化、标准化和集约化。启动实施畜牧业“双千工程”，重点抓好瑞强禽业、金科养殖、大山禽业等30个健康养殖示范企业，新增88个投资千万元以上的标准化规模养殖场。建设全市重大动物疫病防控应急指挥系统，搞好畜禽疫病防治工作。积极推进无公害农产品、绿色食品、有机食品和地理标志产品认证工作，有效提高农业效益和竞争力，率先把我市建成全省高效特色农业示范基地。

　　夯实农业生产基础。围绕山西大水网建设，积极争取吴家庄水库立项，重点抓好沁源永和、屯留屯谷两个应急水源工程，改造15座小型病险水库。完善农机社会化服务体系，农业机械化综合水平达到65%以上。推广保护性耕作和深松作业技术20万亩，改造中低产田20万亩。稳定粮食播种面积，全市粮食总产量保持在14亿公斤左右。

　　启动实施“五大增收工程”。一是设施蔬菜工程，市县两级筹资4亿元对设施蔬菜大棚进行补贴，新建8万亩生贵式移动大棚、2万亩日光式温室大棚，带动农民增收16亿元。二是经济林工程，重点发展核桃、花椒等干果经济林，新增经济林20万亩，改造低效林10万亩。三是规模养殖工程,鼓励发展规模养殖，全年畜禽饲养量要达到3800万头（只）。四是创业就业工程，全年培训农民12万人，新增农民创业就业3.5万人。五是农民专业合作社提升工程，开展合作社联合社、农村资金互助社、土地股份流转合作社“三社”试点工作，再吸纳20%的农户入社，不断增强农民专业合作社的服务带动能力。

　　加快推进新农村建设。实施新农村集中连片建设工程，抓好230个省定重点推进村、300个市整治村和100个中心村建设。大力发展农村公益事业，组织实施新的农村“五个全覆盖”，用两年时间，实现农村街巷硬化全覆盖、农村便民连锁商店全覆盖、农村文化体育场所全覆盖、中等职业教育免费全覆盖、新型农村社会养老保险全覆盖。综合整治村容环境，改善农村生产生活条件。加大扶贫开发力度，抓好定点扶贫工作，加快贫困地区脱贫致富步伐。

　　各位代表，农业安则天下安，农村稳则社会稳，农民富则全民富。我们要以农民增收为核心，投入更多精力、集中更多资源，夯实“三农”基础，让农民得到更多实惠！

　　（四)加快构建“一核双圈”，强力推进市域城镇化

　　加强城市规划和管理。完成《长治市总体规划》评估，编制《上党城镇群体系规划》、《大县城建设规划》、《城南光电园区规划》、《漳泽工业转型试验区战略规划》、《文化传媒创意园区规划》等15个专项规划。加大规划执法力度，严格执行“道路红线”和“绿化绿线”制度，维护城市规划的严肃性。建立统一、协调、高效、合理的城市管理体制，形成政府主导、公众参与、社会监督的城市管理机制。修订完善《长治市拆迁办法》、《长治市土地收储实施办法》。完成数字化城市建设试点任务，建立健全城市智能交通和照明管理信息系统，提高城市精细化管理水平。

增强主城区辐射带动能力。投资168亿元，搞好84个重点项目建设。在新区开发方面，规划建设企业总部园区、行政中心、文化艺术中心、规划展览馆等标志性工程，开工建设市区到新区的通达路，加快新区住宅区建设，搞好供热、供气、供水等公共设施配套工程。在旧城改造方面，全面推进34个城中村和城边村改造，实施“九纵九横”主干道畅通工程和47条支干道微循环畅通工程，加快长安高速市区连接线、北一环、西一环、府后东街、延安北路等11条城市主干道新建改建工程。启动太行东西街景观改造工程，高标准完成客运东站、六府塔公园等工程的绿化，抓好集中供热国电热源市区配套和垃圾处理提档等工程建设。

　　加快上党城镇群内圈建设。高标准建设上党城镇群路网工程，确保国庆节前6条城际连接线通车，做好国省道环城快速通道前期准备，全面提升上党城镇群的通达性、衔接性和承载力。在供气、供水、电力、通信、垃圾污水处理、城乡生态环境、社会事业建设等方面实施一批工程项目，构筑环绕市区的路、电、水、气、通信和绿化六大基础设施网络。今年上党城镇群6个县的建成区面积平均达到5.5平方公里以上，常住人口达到6万人，城镇化率提高2个百分点以上。

　　抓好外圈五个大县城建设。按照“扩大规模、增强实力、完善功能、塑造特色”的要求，规划建设武乡、沁县、沁源、黎城、平顺5个县城，形成外围大县城环带。每个县都要新建或改造3至5项公益性基础设施，发展一批就业容量大的生产性服务业和生活性服务业，扩大县城承载能力，吸引农村剩余劳动力向县城转移、向服务业转移，增加县城人口，让农民进得来、住得下、有钱挣、发展好。今年5个大县城建成区面积提高20%，城镇化率提高1个百分点以上。同时，高起点规划、高标准建设重点镇和中心村，实现城乡一体化发展。

　　各位代表，我市已进入城镇化发展的加速期。我们要把推进城镇化作为今年的一项重要工作，全力攻坚、重点突破，努力建设交通便利、居住宽松、清水绕城、绿树成荫、空气清新、安全放心的一流城镇群！

　　（五）加强生态环境建设，扎实推进城乡生态化

　　狠抓节能降耗。在煤炭、冶金、焦炭、化工、电力、建材等六大高耗能行业继续实施十大节能工程，抓好政策节能、结构节能、工程节能、管理节能。全面开展能效对标活动，加强对100户重点企业的节能监测监管。大力推进建筑节能示范市建设，全面推广绿色环保建筑和节能技术、节能材料，积极倡导低碳消费，促进全社会节能。

　　狠抓治污减排。深入推进蓝天碧水扩容提质工程和上党盆地长治湿地保护，加强重点区域环境综合治理，搞好焦化、冶金等重点用煤行业的脱硫改造，完成所有火电企业脱硫设施建设任务，启动火电行业脱硝工作，加大集中供热锅炉改造力度。提高市区垃圾处理的现代化水平，实施污水处理厂提标升级、脱氮除磷工作，启动污水处理二期工程，确保长北污水处理厂投入运行。加快县城污水处理厂管网配套，污水处理率达到95%以上。

　　狠抓生态建设。以创建国家森林城市、国家生态园林城市为目标，以荒山绿化为重点，高标准完成十大造林绿化工程，确保全年完成营造林40万亩。搞好浊漳河流域综合治理工程、“三河一渠”环城生态水系治理工程和漳泽水库污染治理工程，深入创建国家环保模范城市。

　　各位代表，生态建设和环境保护功在当代、利在千秋。我们要努力建设绿化长治、气化长治、净化长治、健康长治，为全市人民创造一个舒适、怡人的美好空间！

　　（六）切实改善和保障民生，让人民群众共享改革发展成果

　　加快收入分配制度改革。建立企业和机关事业单位职工工资正常增长机制，最低工资标准增长幅度不低于地区生产总值和职工平均工资增长幅度。加快推进事业单位收入分配制度改革，加强对垄断行业的工资监管，促进企业职工工资收入与企业效益同步增长。提高收入分配透明度和公平度，逐步缩小各行业和社会成员之间的收入分配差距，创造条件让更多群众拥有财产性收入。严肃查处压低实际工资、变相违反最低工资制度的行为。

　　扩大社会保障覆盖面。以非公有制经济从业人员、农民工、被征地农民、灵活就业人员和自由职业者为重点，扩大社会保险覆盖面，逐步把城市中稳定就业的农民工纳入社会保险范围。建立低保对象、五保对象保障标准自然增长机制。加快城乡社会救助体系建设，建立临时救助机制。大力推进养老服务社会化。启动城镇居民养老保险试点，新型农村养老保险实现全覆盖。

　　加快保障性住房建设。年内市县两级新建、配建和回购各类保障性住房74.8万平方米、8626套。强化房地产市场监管，抑制房价过快上涨，新建住房价格涨幅控制在城镇居民人均可支配收入增长幅度之内。

　　进一步扩大就业。大力发展就业容量大的劳动密集型企业和生产生活性服务业，把重大投资项目转化为就业岗位。以大学生就业为重点，力争使高校毕业生初次就业率达到70%以上。鼓励创业带动就业，建立两个创业孵化基地，培养2000个创业者，带动1万人就业。积极推行非全日制、临时性、季节性、弹性工作等灵活就业形式，帮助城镇零就业家庭、城乡低保家庭成员实现就业。

　　全面发展各项社会事业。坚持教育优先发展，积极发展学前教育，新建、改扩建144所标准化幼儿园，切实解决幼儿入园难问题；扎实推进义务教育均衡发展，普通高中免学费生比例达到60%以上；大力发展中等职业教育，开工建设长治职业教育园区，中等职业教育免学费比例达到80%。强化科技支撑作用，科技投入达到市级财政支出的1.4%以上，全社会科技研发投入占生产总值比重达到1.2%以上。深化医药卫生体制改革，抓好公立医院改革，全面推广基本药物制度，巩固完善新型农村合作医疗制度，促进公共卫生服务均等化。健全疾病预防控制体系，加强食品药品安全监管。推进上党人口文化“好娃娃”工程，开展免费孕前优生健康检查，促进人口长期均衡发展。积极应对通货膨胀，做好物价异动应对预案，严格落实“菜篮子”、“米袋子”行政首长负责制，不能让物价上涨影响低收入群众的正常生活。

　　毫不松懈抓好安全生产。严格落实政府的安全监管责任和企业的安全生产主体责任，突出抓好装备、素质、管理三大要素，全力推进本质安全型城市创建，今年建成本质安全型矿井10座、安全生产标准化矿井45座。严格执行各项安全生产规定，强化现场管理，夯实安全生产的基层基础工作。深入开展安全生产专项整治，突出抓好煤矿、非煤矿山、尾矿库、道路交通、森林防火、消防、危险化学品、特种设备、易燃易爆物品和公共场所的安全管理。创新事故隐患排查机制，切实做到隐患整改“五到位”、事故处理“四不放过”，努力实现安全生产形势的根本好转。

　　各位代表，活得尊严体面是群众的愿望，改善民生是我们义不容辞的职责。我们要在保障和改善民生上投入更多的人力、物力、财力，把事关老百姓切身利益的事情办实、办好！

　　（七）改革创新、大胆探索，在转型综改试验区建设中先行先试

　　启动转型综改试验区工作。积极申请成为全省国家资源型经济转型综合配套改革试验区的先行区。在建设新兴能源产业基地上先行先试,设立新兴能源产业发展扶持基金，把煤炭可持续发展基金、资源税的留成部分优先用于新兴能源产业基地建设，土地、环保等指标优先保证新兴能源产业项目。在招商引资上先行先试，设立招商引资发展基金，重奖投资引荐人。实行“政策孰优制”，积极开展产业招商、以商招商、以企引企、以资引资，吸引世界500强、国内100强、行业10强来我市投资发展。本市民间资本投资项目，一律享受和外来投资者同样的优惠政策，鼓励民间资本特别是煤矿整合退出资金投入我市重点项目建设。在项目用地上先行先试,制定项目用地单位面积产出率、投资强度、税收贡献、就业安置等控制性指标，对好项目优先供地。盘活存量土地，坚决收回批而不用、长期闲置的土地。整理开发废弃煤矿、尾矿库、堆放场、采石场、农村荒山荒坡以及撤乡并镇、合并学校等空闲土地。鼓励有土地、有厂房的企业与有资金、有技术、有产品的新项目合作。引导农民出村进城、退宅还田，综合开发中心村、城边村，多渠道化解用地矛盾。在建立以煤补林机制上先行先试,从煤炭企业提取生态环境恢复治理保证金，重点用于造林绿化和林业重点工程。在人才保障上先行先试，设立人才引进专项资金，给高层次人才和紧缺人才发放津贴奖励。实行“大学生企官”制度，公开选配优秀大学生到企业工作，除领取企业工资外，地方财政每月补贴500元，在企业工作三年，期间享受大学生村官同等的政治待遇。我市职业技术院校的学生，最后一个学年与企业双向选择签订劳动合同，由企业发放生活补助，毕业后到企业就业。在农民进城上先行先试,凡在城镇有合法固定住所、有稳定职业或生活来源的农民以及公职人员直系亲属等均可转为城镇户口。

加快重点领域和关键环节的改革攻坚。扎实推进行政管理体制改革，强化社会管理和公共服务职能，完善绩效评估和行政问责制度。稳妥推进事业单位分类改革试点。深化财税体制改革，增强基层政府公共服务能力和自身保障能力。加快国有企业改革步伐，依法加大扭亏无望国有企业的关闭破产力度，搞好市潞苑建材、煤炭化工机械厂等31户企业的改制，完善公司法人治理结构，建立现代企业制度。继续推进集体企业改革。创新支持民营经济发展的政策措施，促进民营经济快速健康发展。积极推进投融资体制改革，组建市金融办，支持市商业银行加快发展，再引进几家股份制银行；创新融资方式，支持企业发行中短期融资债券、中小企业集合债，大力支持企业上市直接融资；搭建城市建设投融资平台，争取发行地方政府债券，引导信贷、社会资金参与城镇综合开发。

　　拓宽对外开放的领域和空间。认真做好中博会、高交会、东盟会、华交会等会展招商活动，巩固与德国北威州玛尔市的合作，拓展面向法国、澳大利亚、美国、日本、新西兰和苏丹的全球合作，主动承接东部沿海地区产业转移，加大先进技术、节能环保产品及高端制造业的引进力度。鼓励和支持企业参与全国、全球市场竞争，转变外贸发展方式，巩固传统市场，开拓新兴市场，扩大境外投资和承包工程项目建设。加快筹建长治海关，努力扩大出口规模。

　　各位代表，我们要充分利用转型综改试验区建设这一重大契机，敢行敢试、快行快试、会行会试，靠改革创新增添转型跨越发展的强大动力!

　　（八）加强民主法制和精神文明建设，创造和谐稳定的社会环境

　　加强民主法制建设。自觉接受人大、政协和社会各界的监督，诚恳听取各民主党派、工商联、无党派人士和各人民团体的意见，积极做好人大代表、政协委员建议提案的办理工作。加强基层民主建设，充分保障人民群众的知情权、参与权、表达权、监督权。支持工会、共青团、妇联和科协等群团组织的工作，支持法院、检察院依法独立行使职权。加强政府法制工作，启动“六五”普法，提高全民法制意识。强化审计监督和行政监察，搞好政府系统党风廉政建设。

　　加强精神文明建设。打好文明城市创建攻坚战，以志在必得的信心夺取全国文明城市桂冠。搞好文化基础设施建设，扎实推进文化惠民工程，加快构建覆盖城乡的公共文化服务体系，做好文化遗产保护开发工作。进一步繁荣文化事业，创作一批反映时代风貌、体现长治特色、群众喜闻乐见的精神产品。建设社会主义核心价值体系，大力弘扬太行精神和纪兰精神。加强“信用长治”建设，打造长治信用品牌。

　　加强社会管理。以队伍建设、办公场所建设和强化服务为重点，全面提升城市社区管理水平。畅通群众诉求表达、利益协调、权益保障渠道，做好新形势下的信访工作，对涉及群众利益的重大事项，在决策时要开展信访和社会稳定风险评估。加强社会治安综合治理，深入开展社会矛盾纠纷排查化解。健全突发事件应急管理机制，有效预防和处置各类突发事件。搞好自然灾害预警防范，提高群众防灾减灾的意识和能力。加强网络管理，完善信息发布制度，规范网吧经营行为。支持国防和军队建设，做好双拥工作，推进军民融合式发展。广泛开展全民健身活动，努力提高竞技体育水平。进一步加强统计、对台、档案、保密、民族宗教、外事侨务、人民防空、地方志等工作。关心支持老龄、妇女儿童、残疾人、红十字会、慈善救助等事业。

　　（九）建设人民满意的服务型政府，为转型跨越发展提供坚强保障

　　狠抓落实不懈怠。把各级领导干部的全部心思和精力集中到办实事、抓落实上来，有效解决好事办不好、好事办不快、好事办不了的问题。每一个项目、每一项工作都要锁定责任人，落实到具体环节和岗位，定标准、定进度、定时限，形成市级领导打头阵、县市区领导和市直各部门负责人挑大梁、乡镇长和科长抓落实的责任体系。在各级各部门实行“三个三分之一”工作制度，三分之一的人员外出招商，三分之一的人员在机关办理日常事务，三分之一的人员深入基层、企业提供近距离服务。加大督查力度，采取集中督查、交叉督查、随机督查、限时督办、跟踪督办等方式，“一事一档”建立工作督查台账，定期通报工作进展情况，形成一抓到底的落实机制。

　　狠抓奖惩不动摇。强化对各县市区和市直各部门的考核奖惩力度，鼓励争先创优，严格优胜劣汰，干在前、干得好奖得让人佩服，干得慢、干不好罚得让人信服。对各县市区的考核实行“九奖七罚”，地区生产总值、固定资产投资、工业增加值、财政收入、城镇居民人均可支配收入、农民人均纯收入六项主要经济指标实行严格的百分制考核，90分以上奖励，70分以下主要领导红牌罚出。对市直各部门的考核实行“保六争三”，各项工作要保证在全省排名进入前六名，前三名奖励，第八名以后红牌罚出。

　　狠抓管理不放松。坚持务实高效，转变工作作风，提高行政效能，严查不给好处不办事、给了好处乱办事的行为，对吃拿卡要等现象一经查实，有职务的一律撤职，无职务的一律开除。坚持依法行政，深化政务公开，强化行政监督，规范行政行为，确保公共权力在阳光下运行，提高政府的公信力。坚持廉洁自律，认真贯彻执行廉洁从政各项规定，推进廉政风险防控工作。坚持勤俭节约，把有限的资金更多更好地用在发展经济和改善民生上。

　　各位代表，展望“十二五”美好灿烂的前景，我们豪情万丈、信心百倍。让我们在省委、省政府和市委的坚强领导下，凝聚全市人民的智慧和力量，紧紧抓住综改机遇，勇敢面对各种挑战，解放思想、开拓进取，埋头苦干、奋力争先，为重振上党雄风、再造一个新长治而努力奋斗！
